# Massive splenic cyst in pregnancy: case report

**DOI:** 10.1186/s12884-020-02968-y

**Published:** 2020-05-06

**Authors:** Philip Chung, Ben Swinson, Nicholas O’Rourke, Bart Schmidt

**Affiliations:** 1 Department of Obstetrics and Gynaecology, Royal Brisbane & Women’s Hospital, Brisbane, Queensland Australia; 2 Department of General Surgery, Royal Brisbane & Women’s Hospital, Brisbane, Queensland Australia

## Abstract

**Background:**

Primary splenic cysts are very rarely diagnosed in pregnancy, with only thirteen cases described in the literature. We examine the approach towards diagnosing and managing uniquely large abdominal masses that significantly complicate obstetric care.

**Case presentation:**

A 37-year-old primigravida woman presented with abdominal distension and discomfort, yet otherwise asymptomatic. On ultrasound, an incidental pregnancy at 25 weeks of gestation and a large pelvic lesion were discovered. MRI defined a 28 × 29 cm lobulated, complex cystic mass in the upper abdomen. The patient underwent two ascitic drainages throughout her pregnancy. At 34 weeks of gestation, she had a classical caesarean section. Then at five-weeks postpartum, she underwent a laparotomy and total splenectomy with 16 L of fluid drained. Histopathological analysis revealed an epithelial cyst of the spleen. Her recovery was complicated by complete portal vein thrombosis.

**Conclusion:**

This case describes the largest splenic cyst ever reported in pregnancy and explores the diagnostic dilemmas and treatment challenges associated. We introduce the utility of serial ascitic drainages in prolonging the pregnancy and emphasise the reliance on imaging for surveillance of splenic size and fetal wellbeing.

## Synopsis

A patient with the largest splenic cyst ever reported in pregnancy undergoes caesarean section at 34 weeks, then an open total splenectomy five weeks postpartum.

## Teaching points

1. Magnetic resonance imaging is highly beneficial in defining large abdominal masses in pregnancy especially when splenic cysts are suspected.
2. Careful consideration of patient symptoms, splenic cyst size, and gestational age is advised in counselling patients about timing and mode of delivery, as well as general surgical treatment options.

## Background

True cysts of the spleen are rare clinical encounters with less than 1000 cases reported worldwide. Clinically, splenic cysts are asymptomatic in the majority of cases, otherwise symptoms are non-specific such as vague abdominal discomfort and distension. The management of splenic cysts remains controversial, however traditionally, a surgical approach is advised for symptomatic cysts greater than 5 cm.

We explore the diagnostic dilemma and treatment challenges of a giant primary splenic mass found in pregnancy, a pairing described in only thirteen cases worldwide [1–13]. This case provides further insight into the optimal management for large abdominal masses in pregnancy as the timing and choice of surgical management remains contentious amongst surgeons and obstetricians due to the lack of robust evidence in the current body of literature. Our case presents the largest splenic cyst in pregnancy reported thus far in the literature, and was successfully treated in a major tertiary hospital in Australia.

## Case presentation

A healthy 37-year-old primigravida Australian Indigenous woman presented to a rural Australian hospital with increasing abdominal distension and discomfort, otherwise asymptomatic. She denied recent overseas travels. She reported amenorrhoea for approximately five months. Her past medical, surgical and family history was unremarkable. She was an ex-smoker, reported recreational alcohol intake, and denied any illicit drug use.

On examination, she was obese, and weighed 127 kg. She had a grossly distended, but soft and non-tender, abdomen. Her initial ultrasound scan (USS) showed an incidental intra-uterine pregnancy of 25 + 4 weeks of gestation, cervix long and closed, as well as a complex pelvic lesion with internal septation and vascularity.

**Fig. 1:**
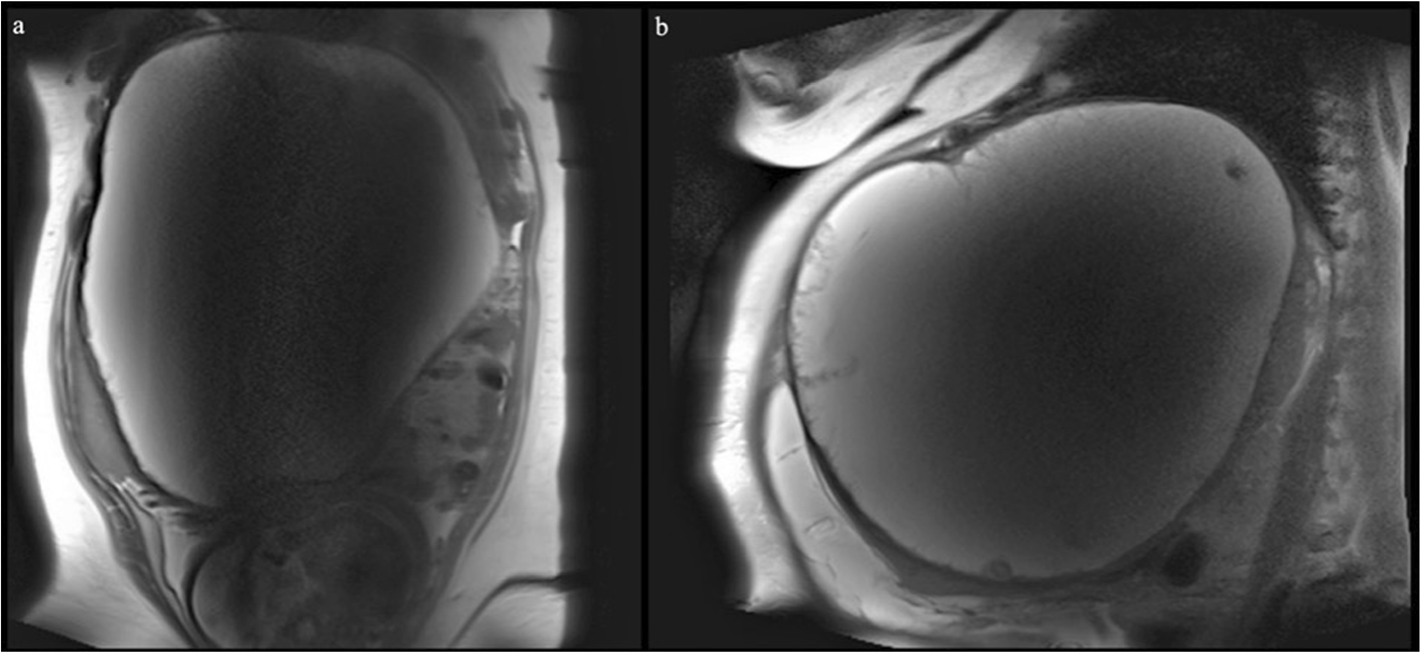
MRI of splenic cyst and fetus. **a** coronial view; **b** sagittal view

The patient was transferred to a major tertiary centre for further investigation and surgical optimisation and planning. Magnetic resonance imaging (MRI) defined a large lobulated, complex cystic mass of unknown origin measuring 28 × 28 × 29 cm in the upper abdomen with nodularity and papillary projections from the wall, and a second 7.0 × 4.6 × 9.8 cm cystic lesion abutting the left kidney with internal septation (Fig. 1). Moderate volume ascites was noted. Her antenatal bloods and biochemistry were unremarkable. Tumour markers were as follows: CEA < 1.0 μg/L (RR < 5.0), CA 19.9120 kU/L (RR < 35), CA 125 11 kU/L (RR < 35), AFP 150 μg/L (RR 30–270), Inhibin B < 10 ng/L (RR < 100–275). ESR 85 mm/hr. (RR < 12), CRP 5.8 mg/L (RR < 5).

At 26 + 1 weeks of gestation, the patient underwent her first ascitic drainage of 1.5 l of cloudy brown fluid, which showed no malignant cells or organisms, and overall features suggestive of an inflammatory reaction. Post-drainage, she had a reassuring pelvic USS at that showed an estimated fetal weight of 1005 g (72nd percentile), with normal AFI and dopplers.

The diagnostic dilemma prompted an Infectious Disease consultation, who tested for *Echinococcus granulosus* serology which came back negative. At 27 weeks of gestation, a chest computed tomography (CT) scan for staging purposes demonstrated enlarged anterior paracardiac lymph nodes, measuring up to 8 mm, however no other lymphadenopathy. Her case was discussed at gynae-oncology multidisciplinary team meeting and the diagnosis was presumed a metastatic ovarian malignancy. Other differential diagnoses included benign ovarian cysts, other abdominal malignancy such as gastric, hepatic or colonic cancer, lymphangioma, infection or inflammation of unknown aetiology, pancreatic abscess or pseudocyst, and splenic enlargement. The preliminary plan was to aim to prolong pregnancy to at least 28 weeks of gestation and steroids 48 h prior to an elective surgery +/− staging.

At 27 + 4 weeks of gestation, the patient underwent a second ascitic drainage of 4 l of cloudy brown fluid, which also showed no malignant cells. A follow-up MRI was performed and demonstrated stable size of the 32 × 27 cm lobulated cystic lesion. At 28 + 1 weeks of gestation, USS showed steady fetal growth 1161 g (33rd percentile). Given the patient had normal fetal movements, reassuring ultrasound scans, and felt symptomatically well, the patient was discharged and offered outpatient follow up. Her USS at 32 + 1 weeks of gestation showed steady fetal growth 1765 g (20th percentile), with normal AFI and dopplers. An obstetric plan for caesarean section at 34 weeks of gestation was subsequently made, taking into careful consideration the risk of potential malignant spread and the patient’s intensifying abdominal discomfort, versus, the risks associated with an early delivery.

With adequate antenatal steroid-loading, at 33 + 6 weeks of gestation, she underwent an elective midline classical caesarean section and exploratory laparotomy with the gynae-oncology team in attendance. As the mass did not appear to arise from the ovaries, Colorectal and Hepatobiliary Surgical teams attended and recommended further imaging before another general surgical procedure. A baby girl was born at 1719 g and discharged in good health after 31 days in the nursery.

**Fig. 2:**
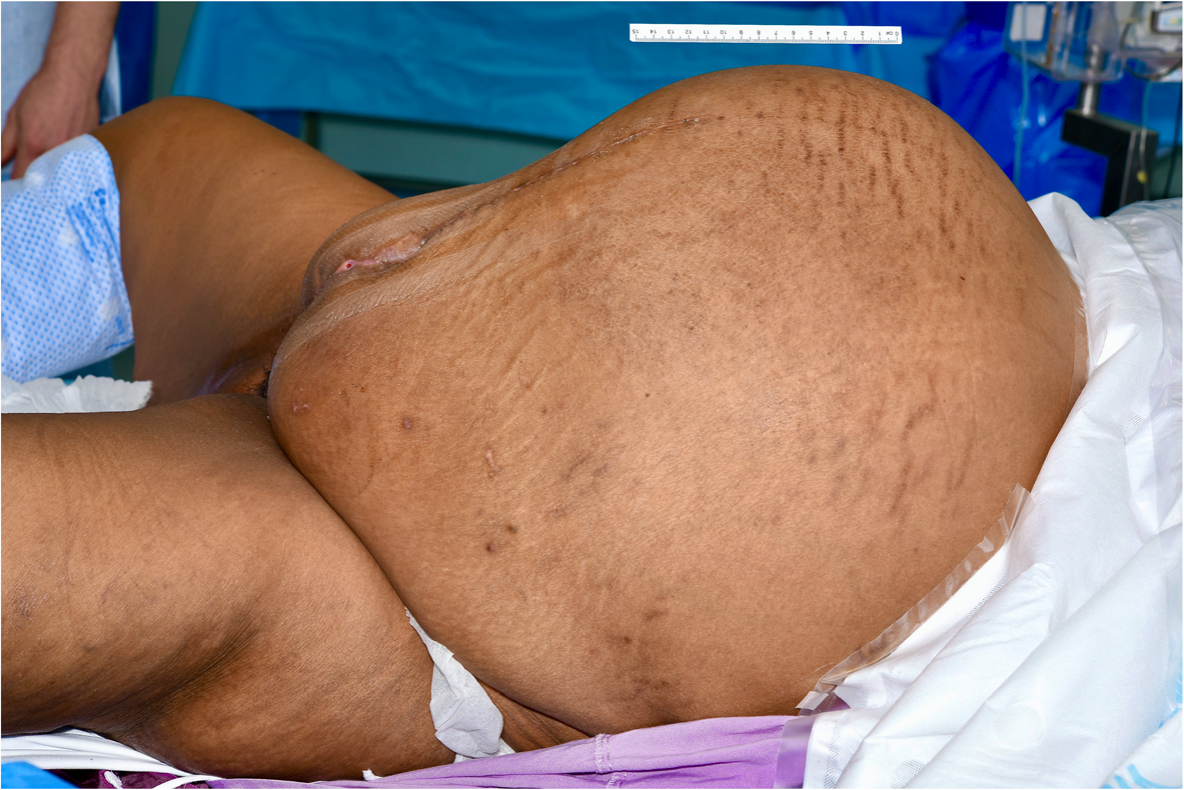
Pre-operative clinical photograph – lateral view of abdominal mass

**Fig. 3:**
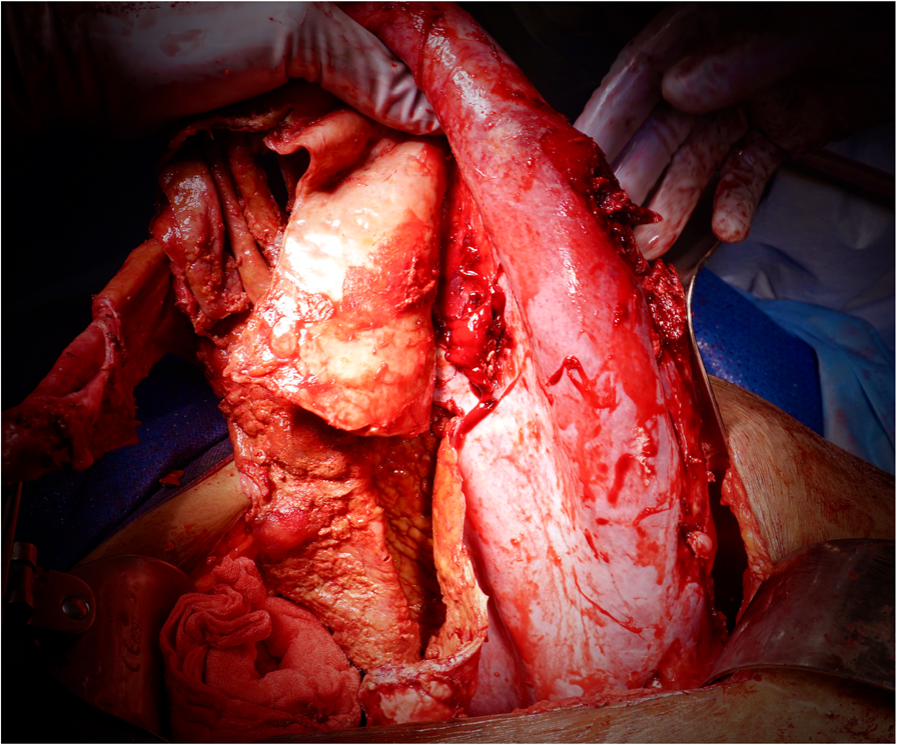
Intra-operative photograph of splenic cyst

**Fig. 4:**
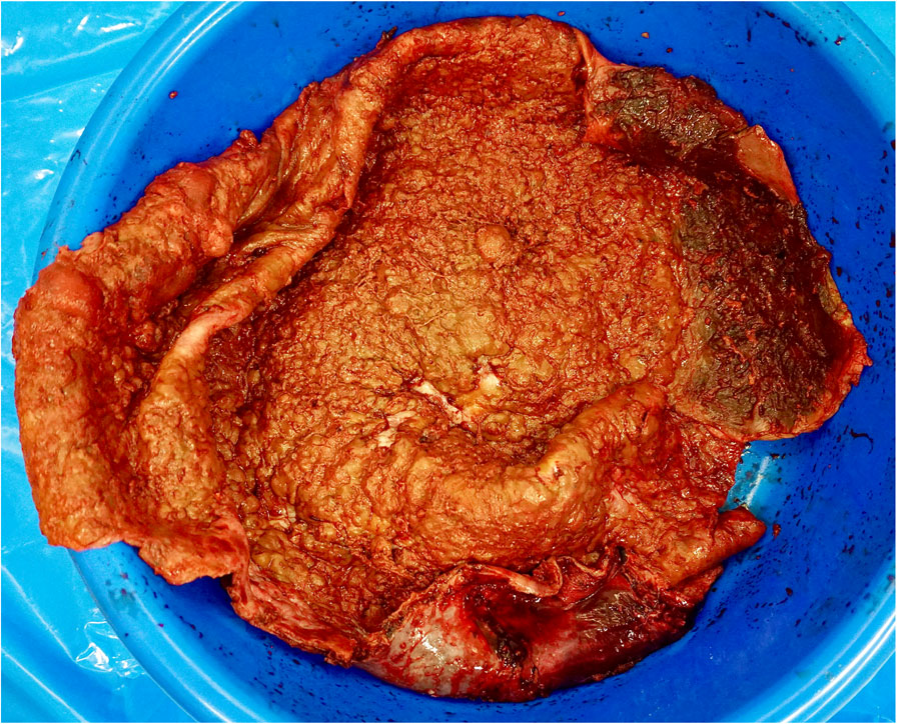
Primary splenic epithelial cyst, measuring 295x155x95mm and weighing 1.3 kg. The cyst wall thickness is 3 mm, outer smooth surface. Inner cyst lining has grey/brown cobblestone appearance

A follow-up CT scan demonstrated a mass originating from the spleen. At five-weeks post-partum, she underwent a midline laparotomy for excision of the splenic cyst (Fig. 2). Initial cystotomy produced sixteen litres of brown turbid fluid. Intra-operative frozen section of the cyst wall confirmed its benign nature, and no bacteria were grown on subsequent culture. The cyst effaced the spleen, with dense attachments to the left hemi-diaphragm and left lobe of the liver. The cyst splayed out the spleen to such an extent that total splenectomy was required (Fig. 3). The spleen was removed with most of the cyst capsule; a small portion of cyst capsule was left due to not being able to safely separate from the diaphragm. The total operative time was 3 h and 30 min. The postoperative course was complicated by portal vein thrombosis that may have arisen from propagation of a pre-existing superior mesenteric vein thrombus that was not well visualised on pre-operative imaging due to severe external compression. Final histological analysis confirmed a primary splenic epithelial cyst (Fig. 4). The patient was discharged three weeks after her operation.

## Discussion and Conclusion

Splenic cysts are rare; the reported incidence rate is 0.07% according to a large case series of 42,327 autopsies over a 25-year period [14]. Fowler [15] and Martin [16] categorise splenic cysts as Type 1 cysts, which are primary (true) cysts with an endocystic epithelial lining, whereas Type II cysts are secondary (false) cysts without epithelial lining. True cysts can be further divided into parasitic (typically *Echinococcus granulosus*) or non-parasitic. Morgenstern [17] subdivides nonparasitic splenic cysts (NPSC) lesions into neoplastic (hemangioma, lymphangioma) and congenital (epidermoid, dermoid, endodermoid). Type II pseudocysts constitute 80% of all splenic cysts, and typically originate from trauma, haemorrhage, abscess, degeneration, inflammation and infarction [18–20]. Hormonal changes in pregnancy may also contribute to splenic infarcts [1].

In terms of demographics, there is a female preponderance [21]; two-thirds of all patients with splenic cysts are aged below 40 [19]. The majority of patients are either asymptomatic or experience minor, non-specific symptoms related to the mass effect of the cyst such as left upper abdominal pain, early satiety, nausea and vomiting [22]. In our case, the patient fits the typical demographic and her late presentation of longstanding abdominal distension with associated discomfort further highlights her stoic character and cultural background as delaying medical attention is not uncommon within the Australian Indigenous population.

Diagnosis of splenic cysts are often incidental during abdominal ultrasonography and can further be defined by CT or MRI scans, in regards to the composition of cystic fluid, the cyst morphology including septations or calcifications, and the surrounding tissue [23, 24]. On ultrasound, primary splenic cysts are smooth, well-defined, and thin-walled cystic lesions with no internal enhancement, whereas secondary splenic cysts appear with thicker fibrous walls and eggshell-like calcifications with debris-related internal echoes [23]. However, internal septations and calcifications may also exist in primary epidermoid splenic cysts [25]. On MRI, splenic cysts depict signal intensities that equal water on both T1 and T2 modalities [26]. Thus, primary splenic cysts and pseudocysts cannot be reliably distinguished solely on radiological findings; histological examination remains the gold standard for ascertaining their aetiology. In pregnant women who require abdominopelvic evaluation, ultrasonography and MRI (without contrast) are the primary imaging modalities recommended; however, CT can be reserved as an alternative as the associated ionising radiation exposure can be reduced to a level (< 50 mGy) such that the effects on the fetus are likely to be negligible at any gestation [27–29].

Furthermore, there is reasonable utility in investigating tumor markers pre- and post-operatively, as the epithelium lining of some primary splenic cysts may produce CA 19.9, CA 125, and CEA [30]. This relationship of raised tumor markers was noted in our case with raised CA 19.9, and similarly demonstrated in previous cases in the literature [31–33]. Though, the authors acknowledge the well-known limitations of elevated tumor markers in pregnancy, the determination of these values as part of the investigative work-up in this case provided meaningful information to the overall clinical picture, especially as the gynae-oncology team were involved in the setting of a large undifferentiated abdominal mass with unclear malignant potential.

A wide range of approaches for splenic cysts in non-pregnant patients have been described, often dependent on patient age, symptoms, cyst characteristics and size. Nonoperative measures such as observation and regular serial imaging is recommended for asymptomatic, small (< 5 cm) cysts [34]. However, for splenic cysts larger than 5 cm, or symptomatic cysts, surgical management is warranted; trials of other conservative methods such as percutaneous aspiration or sclerosis (with either ethanol or tetracycline), though cost-effective with less recovery time, often leads to disease recurrence and may complicate subsequent surgical procedures [30, 35, 36].

Classically, the gold standard surgical approach to splenic cysts has been open total splenectomy. However, due to an evolving understanding of the haematological and immunological functions of the spleen, organ-salvaging techniques have since been developed to mitigate the life-threatening risk of overwhelming post-splenectomy infection (OPSI) [37, 38]. Hence, pre-operative vaccinations for *Streptococcus pneumonia*, *Neisseria meningitides* and *Haemophilus influenza* is crucial, as well as stringent postoperative antibiotic coverage for total splenectomies. Today, the optimal treatment options range from cyst fenestration, marsupialisation and partial cystectomy to total cystectomy with partial splenectomy [39–41].

In pregnancy, the majority of early case reports of splenic cysts underwent splenectomy either open or laparoscopically [1–5]. In later years, two case reports have tried percutaneous aspiration [7, 8]; though both cases had vaginal deliveries at term, both also resulted in cystic fluid re-accumulation and required further intervention. Rotas et al. [7], performed an aspiration for a patient diagnosed with a 17 cm symptomatic splenic cyst in the first trimester, followed by bi-weekly sonographic scans until second trimester. The second cystic aspiration was complicated by sepsis and consequently necessitated laparoscopic fenestration and omentopexy. Likewise, after a conservative approach of iron supplementation, analgesia and antibiotics, Mahran et al. [8] performed a percutaneous aspiration in the second trimester for a 20 cm splenic cyst. However, there were complications of septicaemia and anaemia requiring blood transfusions. Her cystic re-accumulation was noted in the third trimester, but not re-aspirated until six weeks postpartum.

Since then, five more cases of splenic cysts in pregnancy have been reported, of which, four returned to traditional methods of total splenectomy in the second trimester with success. Dabrowski et al. [9] and Forouzesh et al. [11] both describe removal of splenic cysts, measuring 10 cm and 14 cm respectively, via open splenectomy, reporting favourable neonatal outcomes, no postoperative complications and complete resolution of the patient symptoms (pain, anorexia, fever and fullness). Moreover, Majesky et al. [10] and Varban et al. [12] both managed splenic cysts via laparoscopic splenectomy, which also resulted in no postoperative complications and healthy newborns delivered at term. The most recently published case report by Kapp et al. [13] successfully performed a minimally-invasive, organ-preserving procedure of laparoscopic cystectomy for a 15 cm primary splenic epithelial cyst in pregnancy.

In light of these case reports, there appears to be no clear consensus, nor any guidelines, for the management of splenic cysts in pregnancy, as it remains a balance between minimally-invasive approaches of aspiration with risks of re-accumulation and further surgery, versus, organ-preserving approaches of cystectomy, versus, the definitive approach of total splenectomy with greater operative risk and OPSI. Therefore, in counselling obstetric patients about their treatment options, besides general considerations regarding symptoms, cyst size, intraoperative and postoperative risks, additional attention to their gestation, fetal wellbeing, and preference for vaginal delivery or caesarean section is recommended.

In our case, given the patient was in her second trimester at time of presentation, temporising measures such as serial ascitic drainages provided symptomatic relief as we aimed to prolong the pregnancy to an adequate gestation. The exact etiology of the recurrent accumulation of ascitic fluid remains unclear, however the authors postulate its origin from the splenic cyst, and/or, hepatic venous occlusion from the concealed complete portal vein thrombosis owing to the combined compressive effects of the splenic cyst and enlarging gravid uterus. Due to the size of the mass and its compressive effects on surrounding organs, its origin remained unclear despite the use of multiple imaging modalities throughout her pregnancy. This posed a significant diagnostic dilemma that considerably influenced the mode and timing of delivery, as well as the type of general surgical procedure. Ultimately, a splenic mass of such size required an open total splenectomy.

### Strengths and limitations

One of the major strengths in this case presentation is the retrospective analysis of our clinical approach towards diagnosing and managing unusually large abdominal masses in pregnancy. The diagnosis of splenic cyst proved a very unique and unexpected finding for all clinicians involved, hence this case serves as a kind reminder for obstetricians and gynaecologists to consider diagnoses outside of the pelvic region for women presenting with extraordinary abdominal distension. This case presentation not only adds to the limited body of literature on splenic cysts in pregnancy, but also provides insight into an interesting combined case between obstetricians and surgeons where all maternal and fetal treatment options are well considered. The authors advise caution in generalising this rare case report to other more common abdominal masses in pregnancy and acknowledge the advantage of treating such complicated cases in a tertiary hospital where multiple specialties, diagnostic tests and treatment options are readily available.

In conclusion, though epithelial splenic cysts are rare, it ought to be considered as part of the differential diagnoses in young individuals presenting with abdominal distension. We have introduced to the literature the notion of serial ascitic drainages to alleviate patient symptoms and prolong the pregnancy. This case emphasises the reliance on imaging for diagnosis of splenic cysts and serial surveillance of fetal wellbeing. Furthermore, this case highlights the importance of tertiary-level care and implementing a multidisciplinary approach towards complex clinical cases, encouraging good communication between the patient, obstetricians and surgical specialities in exploring all treatment options and optimising maternal and fetal outcomes.

## Data Availability

Not applicable.

## Abbreviations

AFP: Alpha Fetoprotein
CA 125: Carbohydrate antigen 125
CA 19.9: Carbohydrate antigen 19.9
CEA: Carcinoembryonic Antigen
CRP: C-Reactive Protein
CT: Computed Tomography
ESR: Erythrocyte Sedimentation Rate
MRI: Magnetic Resonance Imaging
USS: Ultrasound Scan
